# Circulating microRNA expression signatures accurately discriminate myalgic encephalomyelitis from fibromyalgia and comorbid conditions

**DOI:** 10.1038/s41598-023-28955-9

**Published:** 2023-02-02

**Authors:** Evguenia Nepotchatykh, Iurie Caraus, Wesam Elremaly, Corinne Leveau, Mohamed Elbakry, Christian Godbout, Bita Rostami-Afshari, Diana Petre, Nasrin Khatami, Anita Franco, Alain Moreau

**Affiliations:** 1 Viscogliosi Laboratory in Molecular Genetics of Musculoskeletal Diseases, Office 2.17.027, Sainte-Justine University Hospital Research Center, 3175 Cote-Ste-Catherine Road, Montreal, QC H3T 1C5 Canada; 2 Molecular Biology PhD Program, Faculty of Medicine, Université de Montréal, 2900 Edouard Montpetit Blvd, Montreal, QC H3T 1J4 Canada; 3 Department of Biochemistry and Molecular Medicine, Faculty of Medicine, Université de Montréal, 2900 Edouard Montpetit Blvd, Montreal, QC H3T 1J4 Canada; 4 Open Medicine Foundation ME/CFS Collaborative Center at CHU Sainte-Justine/Université de Montréal, Montreal, Canada; 5 ICanCME Research Network, Sainte-Justine University Hospital Research Center, 3175 Cote-Ste-Catherine Road, Montreal, QC H3T 1C5 Canada; 6 Biochemistry Division, Chemistry Department, Faculty of Science, Tanta University, Tanta, Egypt; 7 Patient-Partner, ICanCME Research Network, Sainte-Justine University Hospital Research Center, 3175 Cote-Ste-Catherine Road, Montreal, QC H3T 1C5 Canada; 8 Department of Stomatology, Faculty of Dentistry, Université de Montréal, 2900 Edouard Montpetit Blvd, Montreal, QC H3T 1J4 Canada

**Keywords:** Biological techniques, Biomarkers

## Abstract

Myalgic encephalomyelitis/chronic fatigue syndrome (ME/CFS), and fibromyalgia (FM) are two chronic complex diseases with overlapping symptoms affecting multiple systems and organs over time. Due to the absence of validated biomarkers and similarity in symptoms, both disorders are misdiagnosed, and the comorbidity of the two is often unrecognized. Our study aimed to investigate the expression profiles of 11 circulating miRNAs previously associated with ME/CFS pathogenesis in FM patients and individuals with a comorbid diagnosis of FM associated with ME/CFS (ME/CFS + FM), and matched sedentary healthy controls. Whether these 11 circulating miRNAs expression can differentiate between the two disorders was also examined. Our results highlight differential circulating miRNAs expression signatures between ME/CFS, FM and ME/CFS + FM, which also correlate to symptom severity between ME/CFS and ME/CFS + FM groups. We provided a prediction model, by using a machine-learning approach based on 11 circulating miRNAs levels, which can be used to discriminate between patients suffering from ME/CFS, FM and ME/CFS + FM. These 11 miRNAs are proposed as potential biomarkers for discriminating ME/CFS from FM. The results of this study demonstrate that ME/CFS and FM are two distinct illnesses, and we highlight the comorbidity between the two conditions. Proper diagnosis of patients suffering from ME/CFS, FM or ME/CFS + FM is crucial to elucidate the pathophysiology of both diseases, determine preventive measures, and establish more effective treatments.

## Introduction

Myalgic encephalomyelitis/chronic fatigue syndrome (ME/CFS) is a disabling, chronic illness with a poorly defined pathophysiology^1^. ME/CFS affects multiple organ systems including immune, neurological, cardiac, endocrine systems, cellular energy metabolism and muscle metabolism, making it a complex disease^2,3^. Key symptoms include debilitating fatigue that is unresolved by sleep, cognitive problems, brain fog and the exacerbation of symptoms following physical or mental activity known as post-exertional malaise (PEM)^4^. Variations in symptoms, severity and duration make the affected population very heterogeneous^1^. In North America, the terms myalgic encephalomyelitis (ME) and chronic fatigue syndrome (CFS) are often used interchangeably, and diagnosis is usually made using the Canadian Consensus Criteria (CCC). The prevalence of ME/CFS is estimated between 0.76 and 3.28% of the population^5^. However, it is also estimated that 84–91% of affected people remain undiagnosed due to complexity, heterogeneity, absence of validated biomarkers and clinical overlaps with other illnesses such as fibromyalgia (FM)^5–7^. FM is another chronic multisystemic condition with an unclear etiology and pathophysiology that affects 2 to 8% of the population, predominantly characterized by chronic pain, low pain threshold, tenderness of muscles, tendons, and joints^8^. Just like ME, the diagnosis of FM is based solely on the clinical criteria due to the lack of validated biomarkers^9,10^. While there are reported differences between the two disorders at the biochemical and molecular levels, these two illnesses have many overlapping symptoms^7,11^. The symptoms of fatigue, sleep problems and cognitive impairment are present in both diseases, which leads to misdiagnosis and up to 34% of comorbidity between the two illnesses^7^. To provide an accurate diagnosis and better understanding of the pathophysiology of both diseases, there is an urgent need to identify novel biomarkers able to discriminate between ME/CFS and FM patients, as well as individuals having a diagnosis of FM associated with ME/CFS (ME/CFS + FM).

One of the promising types of molecules that can serve as good biomarkers are microRNAs (miRNAs). MiRNAs are a class of small non-coding RNAs that regulate gene expression at a post-transcriptional level and play an essential role in developmental and physiological processes^12^. In 2008, Mitchell et al. have established miRNAs as biomarkers for cancer^13^, and since, have been identified as biomarkers for many others diseases^14–18^. We have recently identified a panel of 11 circulating miRNAs (hsa-miR-28-5p, hsa-miR-29a-3p, hsa-miR-127-3p, hsa-miR-140-5p, hsa-miR-150-5p, hsa-miR-181b-5p, hsa-miR-374b-5p, hsa-miR-486-5p, hsa-miR-3620-3p, hsa-miR-4433a-5p and, hsa-miR-6819-3p) associated with PEM and ME/CFS symptom severity, and which could serve as a diagnostic panel for the disease^19^. Interestingly, some of the identified miRNAs, including hsa-miR-29a-3p, hsa-miR-374b-5p and hsa-miR-150-5p have been reported to be dysregulated in FM^20–22^. Once this study established the presence of some altered miRNAs shared between ME/CFS and FM, we explored and compared the expression signatures of a panel of 11 circulating miRNAs in the plasma of ME/CFS, FM, ME/CFS + FM patients and matched healthy controls. We aimed to identify miRNAs that could serve as biomarkers to discriminate between ME/CFS, FM and matched healthy controls, as well as to explore miRNA expression association to symptom severity. The second aim of this study was to demonstrate using a machine learning approach that this panel of 11 circulatory miRNAs can be used to successfully identify patients suffering from ME/CFS, FM or ME/CFS + FM.

## Results

### Clinical and demographic characteristics of participants

**Figure 1:**
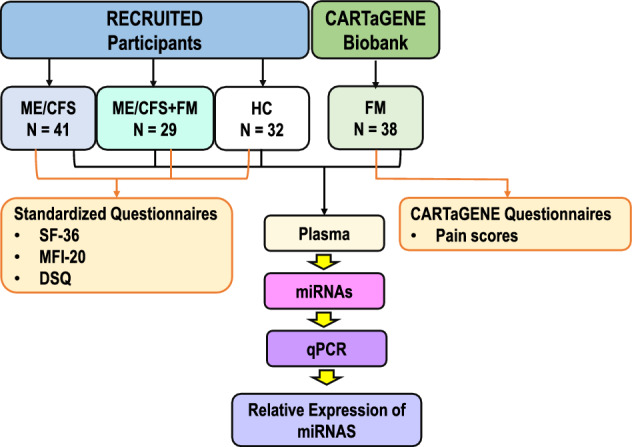
Representation of the experimental study design.

As summarized in our experimental workflow design (Fig. 1), we prospectively enrolled 41 ME/CFS patients (ME/CFS), 29 ME/CFS patients with a comorbid diagnosis of FM (ME/CFS + FM), and 32 matched healthy controls (HC) using the Canadian Consensus Criteria. All recruited participants filled out three standardized questionnaires, Short Form 36-item Health Survey (SF-36), Multidimensional Fatigue Inventory-20 (MFI-20) and DePaul Symptom Questionnaire (DSQ), to assess their quality of life and symptom severity. Plasma samples from 38 individuals with a confirmed diagnosis of FM were obtained from the CARTaGENE biobank^23^. Participants from the CARTaGENE biobank were selected on a basis of a self-reported diagnosis, which was confirmed for most (84%) by an official medical diagnosis made by a rheumatologist, as reported by the *Régie de l'assurance maladie du Québec*, (using the ICD codes 7291 or M797, between the years 1998 to 2021). All the participants were matched in age, ME/CFS and FM were matched in sex, while the ME/CFS + FM participants were only women. No significant difference was observed in age and body mass index (BMI) between ME/CFS, ME/CFS + FM, FM, and HC (Table 1). There was no impact of age or illness duration on the expression of miRNAs (Tables 2, 3). The illness duration for the ME/CFS + FM group included only the duration of ME/CFS illness, since the comorbidity with FM was identified by the answer to question number 86 of the DSQ, “Have you ever been diagnosed with fibromyalgia?”. When comparing the combined scores for different health categories for each self-reported standardized questionnaire, no significant difference was observed between the ME/CFS and ME/CFS + FM participants. However, as expected, there was a significant difference in scores in each health category between ME/CFS and HC and ME/CFS + FM compared to HC (Table 1). Unfortunately, SF-36, MFI-20 and DSQ health questionnaires were not used by CARTaGENE at the time of enrollment. The pain scores for the FM participants were obtained from CARTaGENE health questionnaires variables. A list of self-reported comorbidities of enrolled participants is available in Supplementary Table S1.

**Table 1:** Demographic and clinical characteristics of participants.

	ME/CFS	ME/CFS + FM	FM	HC
N (women:men)	41 (35:6)	29 (29:0)	38 (32:6)	32 (18:14)
Age (years)	53.3 ± 1.2	54.7 ± 1.5	56.7 ± 1.3	50.9 ± 1.7
Illness duration (years)	12 ± 1.8	19.5 ± 2.4	12.3 ± 1.2	N/A
Body mass index (BMI) (kg/m^2^)	25.1 ± 0.7	27.6 ± 1.3	26.9 ± 1.3	25.0 ± 0.8
36-item short-form health survey (SF-36) scores				
Physical score	33.2 ± 2.4****	27.1 ± 2.2****	N/A	90.47 ± 1.4
Mental score	52.7 ± 2.9****	38.8 ± 3.4****	N/A	90.6 ± 1.0
Multidimensional fatigue inventory-20 (MFI-20) scores				
General fatigue	17.6 ± 0.5****	18.7 ± 0.4****	N/A	5.7 ± 0.3
Physical fatigue	17.7 ± 0.4****	18.1 ± 0.5****	N/A	5.8 ± 0.3
Reduced activity	16.0 ± 0.5****	16.9 ± 0.5****	N/A	5.4 ± 0.3
Reduced motivation	9.5 ± 0.5****	10.5 ± 0.5****	N/A	5.6 ± 0.3
Mental fatigue	14.5 ± 0.6****	15.6 ± 0.6****	N/A	6.7 ± 0.5
DePaul symptom questionnaire (DSQ) scores				
Autonomic, neuroendocrine and immune dysfunction	39.9 ± 2.6****	46.3 ± 2.7****	N/A	5.3 ± 0.7
Cognitive dysfunction	55.4 ± 3.1****	60.9 ± 2.8****	N/A	9.7 ± 1.9
Post-exertional malaise (PEM)	69.8 ± 3.3****	76.7 ± 3.1****	N/A	6.3 ± 0.9
Sleep disturbance	51.1 ± 2.8****	55.7 ± 3.5****	N/A	12.9 ± 1.4
The total pain occurrence score	N/A	N/A	3.2 ± 0.3	NA

The health self-reported questionnaires scores for different categories of SF-36, MFI-20, DSQ and the total pain occurrence score. All data are presented as mean and ± standard error of the mean. One-way ANOVA followed by Tukey’s multiple comparisons test were performed to determine the level of the significant difference between the different groups. The questionnaire scores were significantly different between ME/CFS vs. HC and ME/CFS + FM vs. HC. There was no significant difference between ME/CFS and ME/CFS + FM in this data. Results were considered significant at *P value < 0.05, **P value < 0.01, ***P value < 0.001, ****P value < 0.0001.

**Table 2:** Impact of biological sex on symptom severity and miRNA expression.

	ME/CFS		FM		HC	
	Women	Men	Women	Men	Women	Men
36-item short-form health survey (SF-36) scores						
Physical score	30.89 ± 2.13*	47.00 ± 8.98*	N/A	N/A	91.22 ± 2.10	89.50 ± 1.61
Mental score	50.17 ± 3.04*	67.67 ± 7.08*	N/A	N/A	90.44 ± 1.43	90.79 ± 1.27
Multidimensional fatigue inventory-20 (MFI-20) scores						
General fatigue	18.09 ± 0.44*	14.67 ± 2.28*	N/A	N/A	6.22 ± 0.43	5.07 ± 0.31
Physical fatigue	18.14 ± 0.34*	15.00 ± 2.16*	N/A	N/A	6.28 ± 0.48	5.21 ± 0.39
Reduced activity	16.37 ± 0.48	13.83 ± 2.52	N/A	N/A	4.83 ± 0.20	6.07 ± 0.65
Reduced motivation	9.54 ± 0.55	9.00 ± 1.61	N/A	N/A	5.39 ± 0.46	5.79 ± 0.52
Mental fatigue	15.03 ± 0.53*	11.33 ± 2.74*	N/A	N/A	6.78 ± 0.63	6.64 ± 0.79
DePaul symptom questionnaire (DSQ) scores						
Autonomic, neuroendocrine and immune dysfunction	41.97 ± 2.54	27.83 ± 8.91	N/A	N/A	5.44 ± 0.83	5.07 ± 1.26
Cognitive dysfunction	58.23 ± 2.89*	38.83 ± 11.9*	N/A	N/A	7.28 ± 1.46	12.79 ± 3.82
Post-exertional malaise (PEM)	71.46 ± 3.12	60.00 ± 14.2	N/A	N/A	7.39 ± 1.24	4.93 ± 1.29
Sleep disturbance	52.66 ± 2.97	42.17 ± 6.85	N/A	N/A	12.22 ± 1.91	13.71 ± 2.27
The total pain occurrence score	N/A	N/A	3.20 ± 0.85	3.50 ± 0.34	N/A	N/A
MiRNA relative expression						
Hsa-miR-28-5p	2.28 ± 0.59	1.84 ± 0.38	0.08 ± 0.02	0.04 ± 0.03	1.41 ± 0.31	1.32 ± 0.24
Hsa-miR-29a-3p	1.83 ± 0.37	2.11 ± 0.48	0.20 ± 0.03	0.16 ± 0.04	1.09 ± 0.12	1.23 ± 0.23
Hsa-miR-127-3p	3.17 ± 0.62	3.10 ± 1.14	0.19 ± 0.04	0.17 ± 0.13	1.70 ± 0.40	1.31 ± 0.29
Hsa-miR-140-5p	3.17 ± 0.87	2.26 ± 0.78	0.10 ± 0.02	0.02 ± 0.01	1.88 ± 0.55	1.58 ± 0.43
Hsa-miR-150-5p	2.82 ± 0.87	3.92 ± 3.15	0.41 ± 0.09	0.23 ± 0.04	3.31 ± 2.00	3.38 ± 1.70
Hsa-miR-181b-5p	1.73 ± 0.2	1.66 ± 0.22	0.22 ± 0.05	0.10 ± 0.03	1.16 ± 0.25	1.32 ± 0.19
Hsa-miR-374b-5p	3.03 ± 0.72	1.54 ± 0.28	0.12 ± 0.03	0.07 ± 0.04	1.34 ± 0.23	1.19 ± 0.15
Hsa-miR-486-5p	1.25 ± 0.19	2.16 ± 0.78	0.38 ± 0.07	0.31 ± 0.05	1.20 ± 0.21	1.27 ± 0.29
Hsa-miR-3620-3p	1.15 ± 0.21	1.52 ± 0.19	0.13 ± 0.05	0.23 ± 0.22	1.36 ± 0.19	2.07 ± 0.63
Hsa-miR-4433a-5p	1.45 ± 0.24	1.94 ± 0.60	0.14 ± 0.03	0.09 ± 0.04	1.04 ± 0.17	1.86 ± 0.49
Hsa-miR-6819-3p	1.89 ± 0.30	3.95 ± 1.41	0.15 ± 0.05	0.03 ± 0.01	1.23 ± 0.23	1.72 ± 0.36

The health self-reported questionnaires scores for different categories of SF-36, MFI-20, DSQ questionnaires, the total pain occurrence score and the relative expression of the 11 miRNAs of interest. All data are presented as mean and ± standard error of the mean. One-way ANOVA followed by Tukey’s multiple comparisons test were performed to determine the level of the significant difference between the men and women in different groups. Results were considered significant at *P-value < 0.05, **P-value < 0.01, ***P-value < 0.001, ****P-value < 0.0001.

**Table 3:** Impact of age and illness duration on miRNA expression.

miRNA (relative expression)	ME/CFS		ME/CFS + FM		FM		HC
	Age (years)	Illness duration (years)	Age (years)	Illness duration (years)	Age (years)	Illness duration (years)	Age (years)
Hsa-miR-28-5p	*r* = 0.062	*r* = 0.196	*r* = − 0.033	*r* = − 0.070	*r* = 0.017	*r* = 0.069	*r* = − 0.286
	*p* = 0.700	*p* = 0.219	*p* = 0.865	*p* = 0.728	*p* = 0.917	*p* = 0.717	*p* = 0.113
Hsa-miR-29a-3p	*r* = 0.179	*r* = 0.146	*r* = 0.199	*r* = 0.181	*r* = − 0.063	*r* = 0.092	*r* = 0.040
	*p* = 0.262	*p* = 0.364	*p* = 0.300	*p* = 0.367	*p* = 0.706	*p* = 0.630	*p* = 0.830
Hsa-miR-127-3p	*r* = 0.035	*r* = − 0.023	*r* = 0.218	*r* = 0.094	*r* = 0.000	*r* = − 0.056	*r* = − 0.299
	*p* = 0.825	*p* = 0.886	*p* = 0.256	*p* = 0.642	*p* = 0.999	*p* = 0.768	*p* = 0.096
Hsa-miR-140-5p	*r* = 0.136	*r* = 0.137	*r* = − 0.294	*r* = 0.000	*r* = − 0.028	*r* = 0.124	*r* = − 0.262
	*p* = 0.395	*p* = 0.394	*p* = 0.122	*p* = 0.999	*p* = 0.870	*p* = 0.513	*p* = 0.147
Hsa-miR-150-5p	*r* = 0.002	*r* = − 0.087	*r* = − 0.237	*r* = − 0.108	*r* = − 0.061	*r* = − 0.005	*r* = − 0.116
	*p* = 0.989	*p* = 0.588	*p* = 0.216	*p* = 0.591	*p* = 0.717	*p* = 0.981	*p* = 0.527
Hsa-miR-181b-5p	*r* = − 0.120	*r* = − 0.161	*r* = − 0.167	*r* = − 0.254	*r* = − 0.170	*r* = − 0.004	*r* = − 0.173
	*p* = 0.454	*p* = 0.315	*p* = 0.388	*p* = 0.200	*p* = 0.307	*p* = 0.982	*p* = 0.344
Hsa-miR-374b-5p	*r* = 0.136	*r* = 0.043	*r* = 0.177	*r* = − 0.025	*r* = − 0.024	*r* = 0.131	*r* = − 0.061
	*p* = 0.396	*p* = 0.789	*p* = 0.358	*p* = 0.902	*p* = 0.888	*p* = 0.491	*p* = 0.740
Hsa-miR-486-5p	*r* = 0.278	*r* = 0.129	*r* = 0.325	*r* = 0.262	*r* = − 0.115	*r* = 0.014	*r* = − 0.208
	*p* = 0.078	*p* = 0.423	*p* = 0.085	*p* = 0.188	*p* = 0.492	*p* = 0.943	*p* = 0.253
Hsa-miR-3620-3p	*r* = − 0.244	*r* = − 0.161	*r* = 0.141	*r* = 0.292	*r* = − 0.148	*r* = 0.085	*r* = − 0.032
	*p* = 0.125	*p* = 0.314	*p* = 0.465	*p* = 0.139	*p* = 0.375	*p* = 0.655	*p* = 0.863
Hsa-miR-4433a-5p	*r* = 0.066	*r* = − 0.093	*r* = 0.006	*r* = − 0.364	*r* = 0.132	*r* = 0.084	*r* = 0.032
	*p* = 0.680	*p* = 0.562	*p* = 0.976	*p* = 0.062	*p* = 0.429	*p* = 0.658	*p* = 0.861
Hsa-miR-6819-3p	*r* = − 0.073	*r* = − 0.053	*r* = − 0.160	*r* = − 0.174	*r* = − 0.304	*r* = 0.027	*r* = 0.053
	*p* = 0.651	*p* = 0.742	*p* = 0.409	*p* = 0.385	*p* = 0.064	*p* = 0.887	*p* = 0.773

Pearson’s correlation coefficient (r) and the P-values are presented. P values < 0.05 were considered significant.

### Differential miRNA expression signatures between ME/CFS, FM, ME/CFS + FM and HC groups

**Figure 2:**
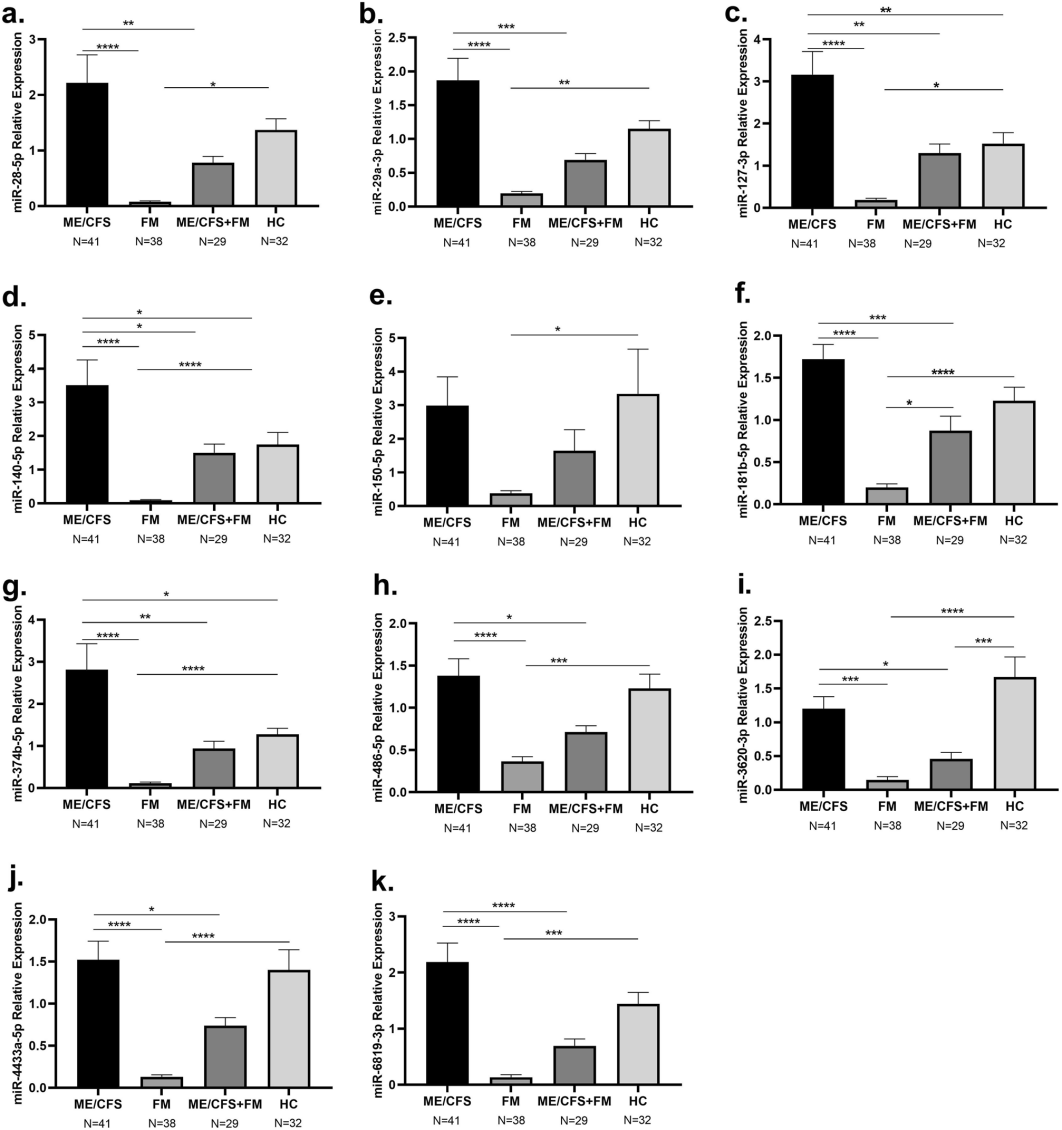
Relative expression of circulating miRNAs in individuals with ME/CFS, FM, ME/CFS + FM and HC. Displayed in the graphs are the mean and ± standard error of the mean of (**a**) hsa-miR-28-5p (**b**) hsa-miR-29a-3p (**c**) hsa-miR-127-3p (**d**) hsa-miR-140-5p (**e**) hsa-miR-150-5p (**f**) hsa-miR-181b-5p (**g**) hsa-miR-374b-5p (**h**) hsa-miR-486-5p (**i**) hsa-miR-3620-3p (**j**) hsa-miR-4433a-5p (**k**) hsa-miR-6819-3p. One-way ANOVA followed by Tukey’s multiple comparisons test were performed to determine the significant difference in the miRNA expression between the groups. Results were considered significant at *P value < 0.05, **P value < 0.01, ***P value < 0.001, ****P value < 0.0001.

The expression of 11 circulating miRNAs, previously associated to ME/CFS^19^, hsa-miR-28-5p, hsa-miR29a-3p, hsa-miR-127-3p, hsa-miR-140-5p, hsa-miR150-5p, hsa-miR181b-5p, hsa-miR374b-5p, hsa-miR-486-5p, hsa-miR3620-3p, hsa-miR4433a-5p and hsa-miR-6819-3p was compared in plasma samples of ME/CFS, FM, ME/CFS + FM and HC patients. We have found that the expression of all tested miRNAs was significantly lower in FM in comparison with HC (Fig. 2a–k), while the expression of miR-127-3p, miR-140-5p and miR-374b-5p was significantly higher in ME/CFS patients compared to HC (Fig. 2c,d,g). In addition, 10 out of 11 miRNAs were differentially regulated between ME/CFS and FM patients. The latter group showed a significantly lower expression of all miRNAs, although the decreased expression of miR-150-5p in the FM group did not reach statistical significance (Fig. 2a–k). Similarly, ME/CFS + FM patients also displayed a reduced expression of all the miRNAs except compared to ME/CFS participants, although miR-181b-5p expression was significantly higher compared to FM and miR-3620-3p expression was significantly lower compared to HC (Fig. 2a–k).

### Pain scores differences between ME/CFS, ME/CFS + FM and HC

**Figure 3:**
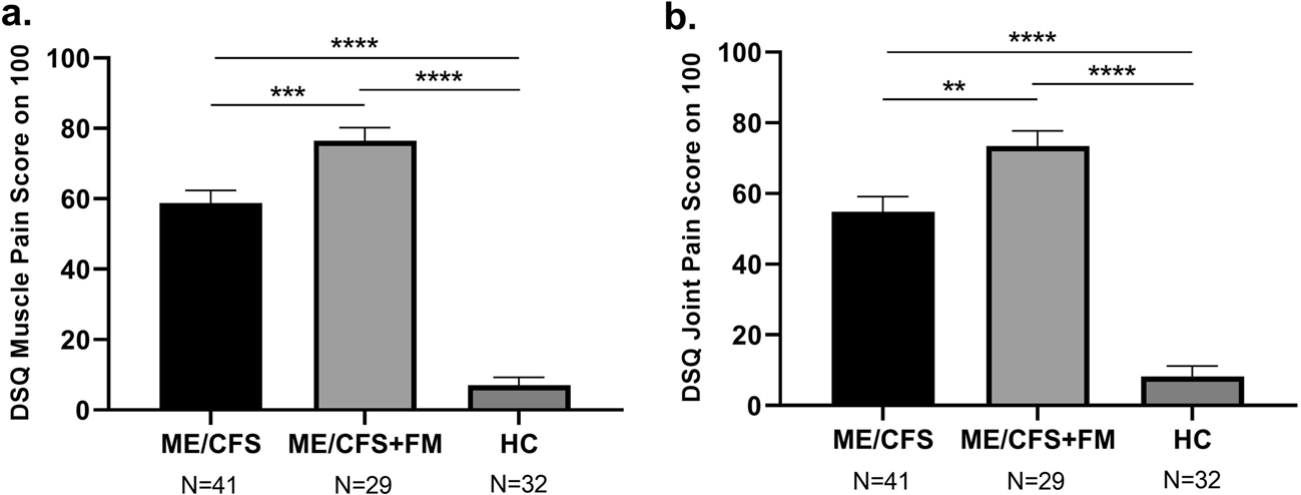
Muscle pain score (**a**) and joint pain score (**b**) from the DSQ questionnaire reported by individuals with ME/CFS, ME/CFS + FM and HC. One-way ANOVA followed by Tukey’s multiple comparisons test were performed to determine the significant difference in the scores between the groups. Results were considered at *P value < 0.05, **P value < 0.01, ***P value < 0.001, ****P value < 0.0001.

Given that muscle pain and joint pain are symptoms predominantly shared by people suffering from FM, we assessed the frequency and severity of muscle pain using question number 25 of the DSQ, and question number 26 to assess joint pain in prospectively enrolled participants. Unsurprisingly, both ME/CFS and ME/CFS + FM groups reported significantly worse pain symptoms in comparison with HC group (Fig. 3a,b). However, we also observed a significant difference in muscle pain scores (p < 0.001) and joint pain scores (p < 0.01) between participants suffering only from ME/CFS and those suffering from both diseases, ME/CFS + FM (Fig. 3a,b). ME/CFS + FM participants report having more frequent and more severe symptoms related to muscle and joint pain when compared to ME/CFS patients.

### Circulating miRNA expression signatures and symptom severity

We investigated if the differential circulating miRNA signatures between ME/CFS and ME/CFS + FM patients were associated with changes in symptoms severity. Search for possible correlations between miRNAs expression and questionnaire scores in ME/CFS patients revealed a negative correlation of miR-150-5p expression level with SF-36 physical score (p < 0.05) while this miRNA correlated positively with DSQ autonomic neuroendocrine immune scores (p < 0.01), PEM scores (p < 0.05), muscle pain (p < 0.05) and joint pain (p < 0.05) scores (Table 4). In addition, expression of miR-3620-3p and miR-6819-3p positively correlated with SF-36 mental score (p < 0.05) while miR-3620-3p negatively correlated with DSQ cognitive scores (p < 0.05) and sleep scores (p < 0.05) (Table 4). When investigating the relationship between miRNA expression and symptom severity in ME/CFS + FM individuals, we observed that miR-150-5p negatively correlated with MFI-20 mental fatigue score (p < 0.05) and with DSQ PEM score (p < 0.01) (Table 4). In addition, we found that miR-374b-5p expression negatively correlated with DSQ sleep score (p < 0.05) and with muscle pain scores (p < 0.05) (Table 4). Moreover, miR-4433a-5p expression negatively correlated with DSQ muscle pain score (p < 0.05). Finally, higher expression of miR-150-5p in FM was positively correlated with higher total pain occurrence (p < 0.04).

**Table 4:** Correlation between 11 miRNAs and different symptoms in ME/CFS, ME/CFS + FM and FM groups.

	SF-36		MFI-20					DSQ						CARTaGENE
	Physical score	Mental score	General fatigue	Physical fatigue	Reduced activity	Reduced motivation	Mental fatigue	Autonomic neuro-endocrine immune	Cognitive	Post exertional malaise	Sleep	Muscles pain	Joints pain	Total pain occurrence
ME/CFS miRNA expression														
miR-150-5p	***r* = − 0.322**	*r* = − 0.014	*r* = 0.206	*r* = 0.146	*r* = 0.013	*r* = 0.043	*r* = 0.198	***r* = 0.416**	*r* = 0.185	***r* = 0.318**	*r* = 0.288	***r* = 0.355**	***r* = 0351**	N/A
	***p* = 0.040**	*p* = 0.932	*p* = 0.196	*p* = 0.363	*p* = 0.937	*p* = 0.790	*p* = 0.215	***p* = 0.007**	*p* = 0.247	***p* = 0.043**	*p* = 0.068	***p* = 0.023**	***p* = 0.024**	
miR-3620-3p	*r* = 0.078	***r* = 0.312**	*r* = − 0.060	*r* = 0.020	*r* = − 0.012	*r* = − 0.116	*r* = 0.070	*r* = − 0.268	***r* = − 0.317**	*r* = 0.034	***r* = − 0.338**	*r* = − 0.076	*r* = − 0.123	N/A
	*p* = 0.627	***p* = 0.047**	*p* = 0.710	*p* = 0.899	*p* = 0.938	*p* = 0.470	*p* = 0.662	*p* = 0.091	***p* = 0.043**	*p* = 0.834	***p* = 0.031**	*p* = 0.636	*p* = 0.443	
miR-6819-3p	*r* = 0.122	***r* = 0.333**	*r* = − 0.143	*r* = − 0.113	*r* = − 0.206	*r* = − 0.269	*r* = − 0.069	*r* = − 0.077	*r* = − 0.199	*r* = − 0.016	*r* = − 0.149	*r* = 0.041	*r* = 0.014	N/A
	*p* = 0.448	***p* = 0.033**	*p* = 0.374	*p* = 0.483	*p* = 0.197	*p* = 0.090	*p* = 0.667	*p* = 0.631	*p* = 0.211	*p* = 0.922	*p* = 0.353	*p* = 0.800	*p* = 0.930	
ME/CFS + FM miRNA expression														
miR-150-5p	*r* = 0.233	*r* = − 0.086	*r* = − 0.065	*r* = − 0.204	*r* = 0.067	*r* = 0.159	***r* = − 0.457**	*r* = − 0.114	*r* = − 0.168	***r* = − 0.521**	*r* = − 0.135	*r* = 0.082	*r* = 0.117	N/A
	*p* = 0.224	*p* = 0.660	*p* = 0.738	*p* = 0.290	*p* = 0.730	*p* = 0.409	***p* = 0.013**	*p* = 0.557	*p* = 0.385	***p* = 0.004**	*p* = 0.484	*p* = 0.672	*p* = 0.547	
miR-374b-5p	*r* = 0.177	*r* = − 0.083	*r* = 0.141	*r* = 0.046	*r* = − 0.013	*r* = 0.151	*r* = 0.333	*r* = − 0.036	*r* = 0.088	*r* = − 0.023	***r* = − 0.401**	***r* = − 0.407**	*r* = − 0.138	N/A
	*p* = 0.358	*p* = 0.669	*p* = 0.466	*p* = 0.812	*p* = 0.948	*p* = 0.436	*p* = 0.078	*p* = 0.854	*p* = 0.648	*p* = 0.904	***p* = 0.031**	***p* = 0.028**	*p* = 0.475	
miR-4433a-5p	*r* = 0.331	*r* = 0.283	*r* = 0.015	*r* = − 0.05	*r* = − 0.255	*r* = 0.123	*r* = − 0.126	*r* = − 0.113	*r* = − 0.030	*r* = − 0.345	*r* = − 0.329	***r* = − 0.436**	*r* =− 0.090	N/A
	*p* = 0.08	*p* = 0.137	*p* = 0.940	*p* = 0.797	*p* = 0.182	*p* = 0.525	*p* = 0.514	*p* = 0.559	*p* = 0.878	*p* = 0.067	*p* = 0.082	***p* = 0.018**	*p* = 0.641	
FM miRNA expression														
miR-150-5p	N/A	N/A	N/A	N/A	N/A	N/A	N/A	N/A	N/A	N/A	N/A	N/A	N/A	***r* = *0.33***
														***p* = 0.04**

Pearson’s correlation coefficient (r) and the P-value are presented. P values < 0.05 were considered significant and are indicated in bold.

### ME/CFS vs FM differential diagnosis according to machine-learning approach

**Figure 4:**
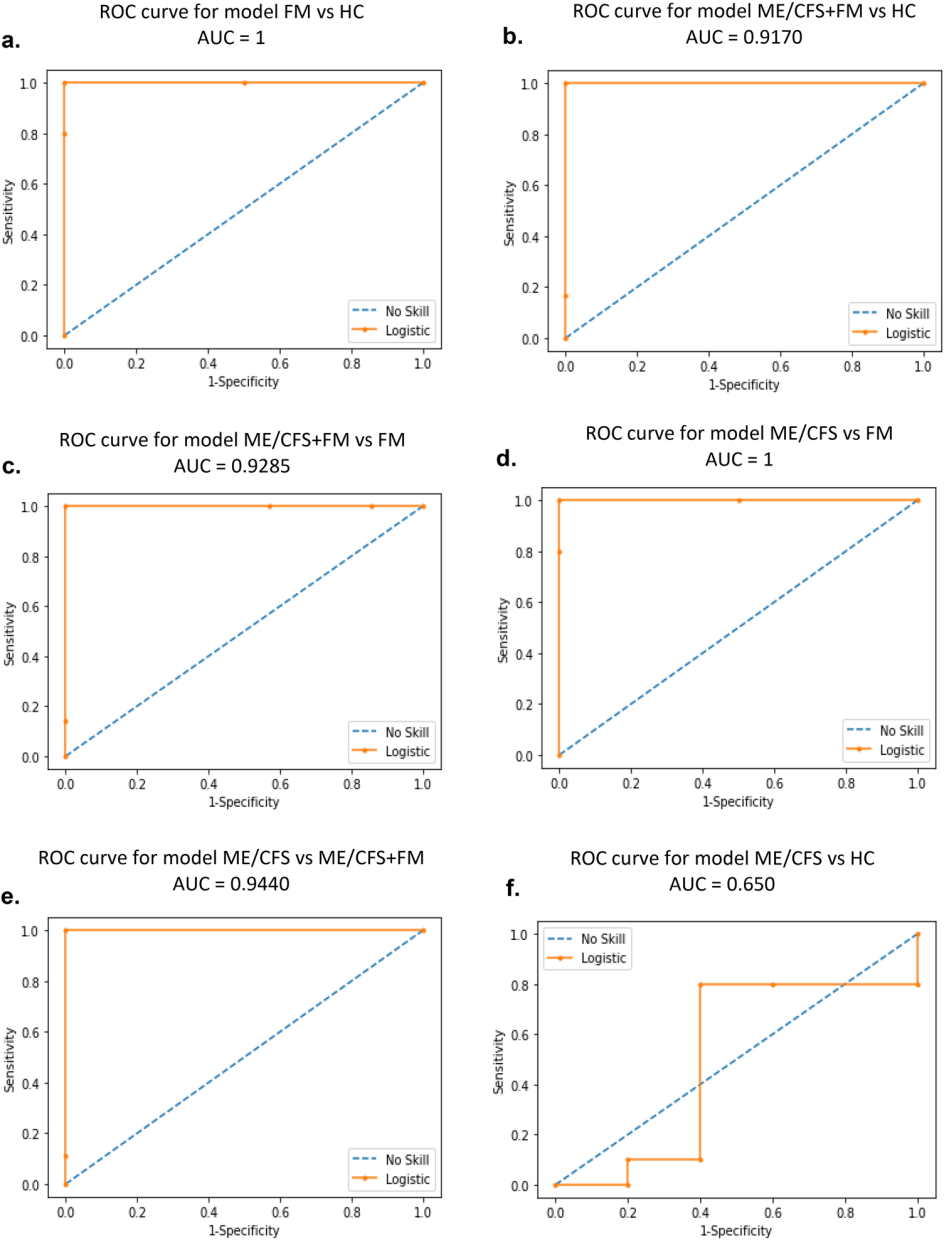
ROC curves for different prediction models using Random Forest Model. (**a**) ROC curve for prediction model classifying FM versus HC. (**b**) ROC curve for prediction model identifying ME/CFS + FM versus HC. (**c**) ROC curve for prediction model for classification of ME/CFS + FM versus FM. (**d**) ROC curve for prediction model identifying ME/CFS versus HC. (**e**) ROC curve for prediction model classifying ME/CFS versus ME/CFS + FM. (**f**) ROC curve for prediction model identifying ME/CFS versus HC.

Random Forest Models (RFM) were developed to discriminate individuals with symptoms commonly observed in ME/CFS and FM in a specific disease group using the expression profiles of 11 circulating miRNAs. We initially applied RFM to the 2^−ΔΔCT^ data to a training dataset representing 80% of our patient cohorts. We then evaluated the performance of our models on a testing dataset corresponding to 20% of our cohort. We generated receiver operating characteristic curves (ROC curves) and obtained a specificity and sensitivity of 100% with and area under the curve (AUC) of 1 (Fig. 4a) when classifying FM patients versus HC. When applying the RFM to the ME/CFS + FM and HC dataset, we obtained a specificity of 100% and a sensitivity of 83% with ROC curve AUC of 0.9170 (Fig. 4b). When classifying ME/CFS + FM versus FM, we obtained a specificity of 100% and a sensitivity of 86% with a ROC curve AUC of 0.9285 (Fig. 4c). When applying the model to the ME/CFS and FM datasets, we again reached a specificity and sensitivity of 100% with ROC curve AUC of 1 (Fig. 4d). To discriminate between ME/CFS patients versus ME/CFS + FM individuals, we obtained a model with a specificity of 100%, a sensitivity of 89% with a ROC curve AUC of 0.9440 (Fig. 4e). However, the prediction model based on the ME/CFS and HC datasets only reached a specificity of 60%, a sensitivity of 70% with a ROC AUC of 0.650 (Fig. 4f).

## Discussion

ME/CFS and FM are two complex chronic diseases with unknown etiology, classified with different codes by the International Classification of Diseases, Tenth Revision, Clinical Modification (ICD-10-CM)^24,25^. Despite overlapping symptoms, the distinction between the two diseases remains under debate^11,26^. Due to the lack of specific biomarkers, the diagnosis of both diseases maybe delayed and often results through a lengthy exclusion process to eliminate other diseases causing similar symptoms^27,28^. According to McKay et al*.* some individuals diagnosed with one disease often meet the criteria for the other, which can lead to a potential misdiagnosis while the presence of ME/CFS with a comorbid diagnosis of FM is often overlooked^26^. Nevertheless, many studies provide evidence that ME/CFS and FM are indeed two separate illnesses with distinct underlining etiology^29–31^. In this study, the discovery of differential circulating miRNA expression signatures associated with ME/CFS, FM and ME/CFS + FM groups further supports that ME/CFS and FM are two distinct related illnesses.

Our study replicated for the first time in the French-Canadian population, the association between FM and the decreased expression of circulating miR-29a-3p, miR-150-5p and miR-374b-5p as previously reported in other populations^20–22^. Indeed, Bjersing JL et al*.* reported a significantly decreased expression of miR-29a-3p and miR-374b-5p respectively in cerebrospinal fluid and serum of FM patients compared to HC^20,21^. Interestingly they showed that miR-374b-5p expression negatively correlates with pain threshold^21^. Notwithstanding methodological differences in the quantification of pain between studies, we also observed a significant negative correlation between miR-374b-5p expression levels and DSQ muscle pain scores. Intriguingly, this negative correlation occurred only in ME/CFS + FM patients and not in the ME/CFS group. This result further supports the implication of miR-374b-5p in the enhanced pain perception observed in ME/CFS + FM patients, which is most likely mediated by the co-occurrence of FM as comorbidity. Buron et al. recently designed a selective discriminatory algorithm using available microarray datasets from FM patients^22^. They found that miRNA datasets show statistically higher accuracy in classifying FM patients from HC than mRNA expression datasets and that miR-150 and miR-29a, which were both down-regulated, were part of a panel of 20 miRNAs that yielded the best accurate results^22^. Our data confirm the downregulation of miR-29a-3p and miR-150-5p in our FM patients compared to HC.

We compared the expression profiles of ME/CFS, ME/CFS + FM and FM patients to get better insight into the disease-specific deregulations in the expression of different miRNAs compared to the healthy controls. Among the 11 miRNAs, miR-127-3p, miR-140-5p and miR-374b-5p were over-expressed in ME/CFS and under-expressed in FM. These three miRNAs could be used as potential biomarkers to distinguish ME/CFS from FM.

Using a machine-learning approach, RFM models integrating the differential expression signatures of 11 circulating miRNA were successfully developed to discriminate ME/CFS, FM, ME/CFS + FM and HC groups. However, the prediction model based of ME/CFS versus HC was not sensitive or specific enough for this task. This result is not surprising since we have previously demonstrated a prediction model based on the differential expression of this panel of 11 miRNAs in response to the application of a stress test inducing PEM in ME/CFS patients when compared at baseline^19^.

In the present study, we observed the lower miRNAs expressions of miR-3620-3p and miR-6819-3p in ME/CFS, or miR-150-5p, miR-374b-5p and miR-4433a-5p in ME/CFS + FM, were associated with more severe symptoms scores. Contradictory, the higher miR-150-5p expression was correlated with higher PEM scores in ME/CFS and total pain occurrence in FM. This underlines the difference between ME/CFS, ME/CFS + FM and FM diseases and the importance of recognizing the comorbidity between the two illnesses.

Based on our results, miR-150-5p is down-regulated in FM and associated with symptom severity in ME/CFS. Several studies showed that miR-150-5p might appear as a central regulator of gene expression during the immune cell differentiation and immune response process. Also, it plays a vital role in inhibiting B cell activation and differentiation and regulates the cellular immune defense against invading pathogens^32^. Dysregulated expression of miR-150-5p in immune cells could result in autoimmune diseases^33^. These explanations support the hypothesis that ME/CFS is may be an autoimmune disease^34^.

**Figure 5:**
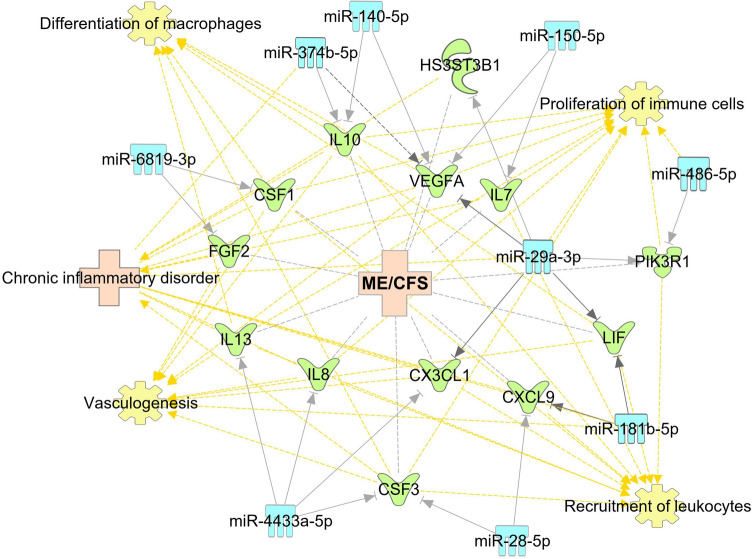
Genes related to ME/CFS that are predicted or confirmed targets of the 11 miRNAs. The miRNAs are presented in light blue. The targets of miRNAs are in green, ME/CFS, FM and other related diseases are in light pink, and associated functions of genes are in yellow.

**Figure 6:**
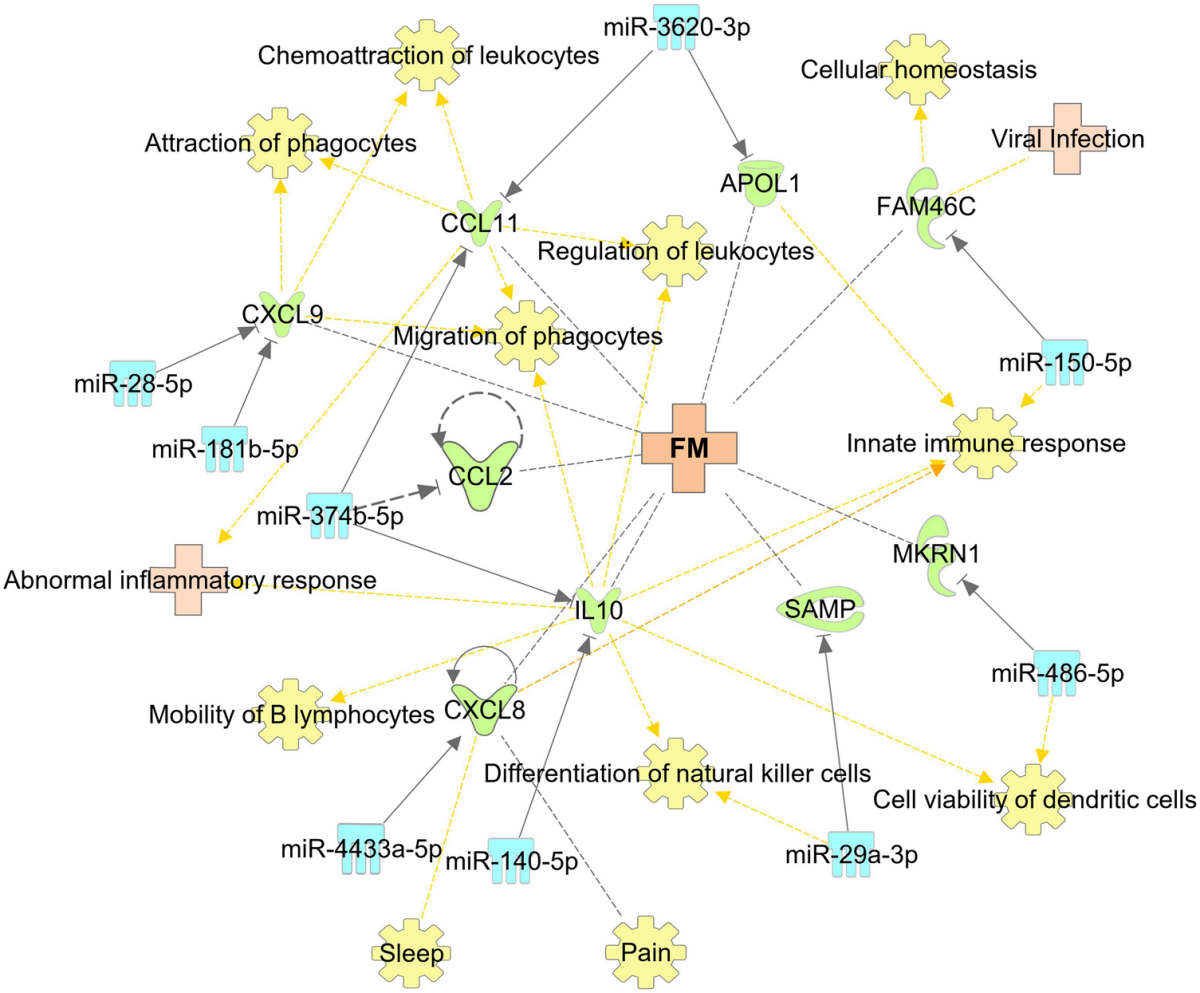
Genes related to ME/CFS or FM that are predicted or confirmed targets of the 11 miRNAs. The miRNAs are presented in light blue. The targets of miRNAs are in green, ME/CFS, FM and other related diseases are in light pink, and associated functions of genes are in yellow.

In addition to miR-150-5p, other identified miRNAs can play a role in the deregulation of immune system and other dysfunctions observed in ME/CFS and FM. It has been reported that levels of a proinflammatory cytokine IL-8, chemokine CXCL9 and anti-inflammatory IL-10 are reported to be elevated in biological fluids of FM patients, and in contrast are seen to be decreased in individuals with ME/CFS^35,36^. Interestingly, we observe significantly reduced levels in FM and elevated levels in ME/CFS of circulatory miRNAs that can regulate the expression of those genes. According to our pathway analysis, IL-8 gene transcript is a predicted target of miR-4433a-5p (Figs. 5, 6), and this cytokine has been reported to play a role in sleep regulation and found to be related to pain intensity in FM patients^37,38^. CXCL9 participates in the regulation of immune cell migration, differentiation, and migration^39^, is a predicted target of miR-181b-5p and miR-28-5p (Fig. 5, 6). MiR-140-5p and miR-374b-5p are predicted to target the IL-10 gene (Fig. 5, 6), an important cytokine that is secreted by almost all cells of the innate and adaptive immune systems that dampers Th1 immune related responses^40^.

Eotaxin-1, encoded by CCL11 gene is reported to be elevated in the plasma of FM^41^ and is a predicted target of miR-374b-5p and miR-3620-3p (Fig. 6). Eotaxin-1 is an important chemokine that is associated with recruitment of eosinophils to the site of inflammation^42^. MiR-374b-5p is also predicted to target CCL2 gene that codes for MCP-1 which has also been reported to be elevated in FM^41^ (Fig. 6). It was proposed that the elevation of MCP-1 may be related to abnormalities in energy metabolism found in biopsy specimens of tender areas of FM patients because MCP-1 was shown to reduce insulin-stimulated glucose uptake in skeletal muscles^41,43^.

One of the confirmed targets of miR-374b-5p is the transcript of VEGFA^44^ (Fig. 5). The levels of VEGF-A protein are reported to be reduced in the plasma of ME/CFS patients^45^. VEGF-A could play a role in post-exertional malaise and fatigue experienced by ME/CFS patients because VEGF-A is known to direct vasculogenesis and angiogenesis, plays a role in the maintenance of capillary supply in normal skeletal muscle and its mRNA was shown to increase in skeletal muscles following acute exercise^45,46^.

Overall, the identification of altered miRNAs in both ME/CFS and FM and the definition of the opposite patterns of expression may overlay the way for new studies to better elucidate the involvement of these miRNAs in ME/CFS and FM. Further studies are required to examine the influence of these miRNAs on different pathways and physiological processes, as described in Figs. 5 and 6. The present study results provide a non-invasive diagnostic biomarker for ME/CFS and FM based on the expression profile of circulating miRNAs.

Among possible limitations, longitudinal studies must be undertaken to evaluate the implication of miRNA in disease progression in the context of ME/CFS, FM and ME/CFS + FM. In addition, further miRNA target validation should be undertaken and possible impact on patient health status by treatments regulating miRNA expression should be explored.

In conclusion, the present study identified 11 miRNAs found to be altered in FM and ME/CFS. In particular, we analyzed the potential involvement of these miRNAs in the onset of both diseases, although oppositely expressed. For the first time, to our knowledge, we provided evidence showing that miRNA expression levels miR-127-3p, miR-140-5p and miR-374b-5p could be potential biomarkers for ME/CFS and FM illnesses. Using a machine learning approach based on the panel of 11 circulatory miRNA relative expressions, we successfully discriminated between ME/CFS, FM and ME/CFS + FM, as well as FM and ME/CFS + FM against healthy controls. Finally, the results of our study might help diagnose either disease by distinguishing the two conditions.

## Materials and methods

### Study populations

Forty-one individuals diagnosed with ME/CFS, twenty-nine diagnosed with both ME/CFS and FM (ME/CFS + FM), and thirty-two age-matched sedentary healthy controls were recruited for this study. In addition, plasma of thirty-eight FM patients was obtained from CARTaGENE biobank (Table 1). ME/CFS were diagnosed using the Canadian consensus criteria. FM diagnosis was established by rheumatologists through a complete evaluation of medical history and a full physical exam. Question number 86 of the DSQ, “Have you ever been diagnosed with fibromyalgia?” established the comorbidity of FM and ME/CFS. The healthy control subjects had no family history or symptoms of ME/CFS or FM. The protocol of this study was approved by the Institutional Review Board of Sainte-Justine University Hospital (protocol #4047). All participants provided written informed consent. All experiments were performed following relevant guidelines and human ethic regulations.

### Evaluation and quantification of symptoms and recruited participant health status

All recruited participants completed standardized questionnaires to assess their health status and symptom severity. The questionnaires included the 36-Item Short-Form Health Survey (SF-36), Multidimensional Fatigue Inventory (MFI-20) and the DePaul Symptom Questionnaire (DSQ). These questionnaires provide information on several health categories. SF-36 scale provides physical and mental health scores^47^. MFI-20 questionnaire scores are combined to assess general fatigue, physical fatigue, reduced activity, reduced motivation, and mental fatigue^48^. DSQ questions were grouped into four categories: neuroendocrine, autonomic and immune dysfunction, cognitive dysfunction, post-exertional malaise, and sleep disturbances^49^. Questions 25 and 26 of the DSQ were used to respectively quantify muscle and joint pain. During recruitment, all participants were asked to disclose any health conditions. FM pain scores were obtained from CARTaGENE health questionnaire variables.

### Blood specimen collection, small RNA extraction, complementary DNA (cDNA) synthesis and qPCR analysis

The collection of blood samples from the recruited participants, plasma preparation, small RNA extraction from plasma, cDNA synthesis and miRNA detection by qPCR was done as previously described without modifications^19^. The FM plasma samples were obtained from CARTaGENE biobank. Blood collection from both the recruited participants and the CARTaGENE biobank were similarly performed, using EDTA-K2 collection tubes and in non-fasting conditions. In both cases, specimens were stored at − 80 °C until analysis.

### qPCR data quantification

The relative miRNA expression in each sample was quantified using the 2^−ΔΔCT^ method as previously described^19^.

### Construction of gene pathways and networks targeted by dysregulated miRNAs in ME/CFS and FM

Genes associated with either ME/CFS or FM and the predicted targets of the 11 miRNAs of interest were identified using Ingenuity Pathway Analysis (IPA) software (QIAGEN Inc. software version 70,750,971).

### Machine learning and statistical analysis

Machine learning method, Random Forest Model (RFM) was used to construct models that could be used to predict and differentiate between FM and HC, ME/CFS + FM and HC, ME/CFS + FM and FM, ME/CFS and FM, ME/CFS and ME/CFS + FM, ME/CFS and HC. For each prediction model, the data were randomly separated into the training dataset, representing 80% of the data and the testing dataset, the remaining 20%. RFM model was built using the training dataset and tested on the testing dataset. To evaluate the RFM, the specificity, sensitivity, and receiver operating characteristics (ROC) curve was used to determine the classification performance. The ROC curves presented the trade-off between the sensitivity and specificity in each model, and the area under the curve (AUC) was used to evaluate its predictive performance. MiRNAs expression data results, questionnaire score results and other clinical characteristic data were presented as mean ± SEM; a significant difference in data was determined using one-way ANOVA followed by Tukey multiple comparison test. The correlation between miRNA expression and questionnaire scores was analysed using Pearson correlation. The differences between men and women in questionnaire scores and miRNA expression was analysed using student’s *T* test. P-values smaller than 0.05 were considered significant. Statistical analysis was performed using GraphPad Prism (version 8, GraphPad Software, Inc., San Diego, CA, United States).

## Supplementary Information

Supplementary Table S1.

## Data Availability

The datasets that were generated and that were used for analysis for this study are available through the corresponding author.
