# Supplementary material for: Circulating microRNA expression signatures accurately discriminate myalgic encephalomyelitis from fibromyalgia and comorbid conditions
Source: Sci Rep. 2023 Feb 2;13:1896. doi: 10.1038/s41598-023-28955-9 (PMC9894933; doi:10.1038/s41598-023-28955-9)
Supplement: Supplementary file 1 — Supplementary Table S1. [file 41598_2023_28955_MOESM1_ESM.docx]

**Circulating MicroRNA Expression Signatures Accurately Discriminate Myalgic Encephalomyelitis from Fibromyalgia and Comorbid Conditions**

Evguenia Nepotchatykh^1,2,4,5^, Iurie Caraus^1,3,4,5^, Wesam Elremaly^1,4,5^, Corinne Leveau^1,3,4,5^, Mohamed Elbakry^1,4,5,6^, Christian Godbout^7^, Bita Rostami-Afshari^1,3,4,5^, Diana Petre^1,3,4,5^, Nasrin Khatami^1,3^, Anita Franco^1,4,5^, and Alain Moreau^1,3,4,5,8,*^

^1^Viscogliosi Laboratory in Molecular Genetics of Musculoskeletal Diseases, Sainte-Justine University Hospital Research Center, 3175 Cote-Ste-Catherine Road, Montreal, QC, H3T 1C5, Canada;

^2^Molecular Biology PhD Program, Faculty of Medicine, Université de Montréal, 2900 Edouard Montpetit Blvd, Montreal, QC, H3T 1J4, Canada;

^3^Department of Biochemistry and Molecular Medicine, Faculty of Medicine, Université de Montréal, 2900 Edouard Montpetit Blvd, Montreal, QC, H3T 1J4, Canada;

^4^Open Medicine Foundation ME/CFS Collaborative Center at CHU Sainte-Justine/Université de Montréal;

^5^ICanCME Research Network, Sainte-Justine University Hospital Research Center, 3175 Cote-Ste-Catherine Road, Montreal, QC, H3T 1C5, Canada;

^6^Biochemistry Division, Chemistry Department, Faculty of Science, Tanta University, Tanta, Egypt;

^7^Patient-partner, ICanCME Research Network, Sainte-Justine University Hospital Research Center, 3175 Cote-Ste-Catherine Road, Montreal, QC, H3T 1C5, Canada;

^8^Department of Stomatology, Faculty of Dentistry, Université de Montréal, 2900 Edouard Montpetit Blvd, Montreal, QC, H3T 1J4, Canada;

^*^Corresponding Author: Alain Moreau, Viscogliosi Laboratory in Molecular Genetics of Musculoskeletal Diseases, Office 2.17.027, Sainte-Justine University Hospital Research Center, 3175 Cote-Ste-Catherine Road, Montreal, QC, H3T 1C5, Canada. Tel.: 514-345-4931 ext 5722,

[alain.moreau.hsj@ssss.gouv.qc.ca](mailto:alain.moreau.hsj@ssss.gouv.qc.ca)

**SUPPLEMENTARY INFORMATION**

**Supplementary Table S1.** List of self-reported comorbidities of the participants

|  | Number of participants in each group | | | |
| --- | --- | --- | --- | --- |
| Diagnosed with or treated for: | ME/CFS  **N=41** | ME/CFS+FM  **N=29** | FM  **N=38** | HC  **N=32** |
| Allergies | 15 | 15 | 0 | 5 |
| Amblyopia | 0 | 0 | 1 | 0 |
| Anemia | 0 | 1 | 12 | 0 |
| Ankylosing spondylitis | 0 | 0 | 1 | 0 |
| Anxiety | 12 | 10 | 6 | 2 |
| Arthritis | 0 | 0 | 15 | 0 |
| Asthma | 3 | 0 | 3 | 1 |
| Atherosclerosis | 0 | 0 | 1 | 0 |
| Atrophic lichen sclerosus | 1 | 0 | 0 | 0 |
| Attention deficit disorder | 1 | 0 | 0 | 0 |
| Bipolar disorder | 0 | 0 | 0 | 1 |
| Bladder infection | 0 | 0 | 7 | 0 |
| Breast cancer | 0 | 0 | 2 | 0 |
| Cataract | 0 | 0 | 4 | 0 |
| Celiac disease | 1 | 1 | 1 | 0 |
| Cervical cancer | 0 | 0 | 3 | 0 |
| Cervical spondylosis | 1 | 0 | 0 | 0 |
| Chlamydia | 0 | 0 | 1 | 0 |
| Cholesterol | 1 | 0 | 11 | 2 |
| Chronic Bronchitis | 0 | 0 | 3 | 0 |
| Chronic ear infection | 0 | 0 | 2 | 0 |
| Color vision problems | 0 | 0 | 2 | 0 |
| Diverticular Disease | 0 | 0 | 4 | 0 |
| Eating disorder | 1 | 1 | 2 | 0 |
| Eczema | 0 | 0 | 12 | 1 |
| Endometriosis | 0 | 0 | 5 | 0 |
| Epilepsy | 0 | 0 | 2 | 0 |
| Fatty liver | 0 | 0 | 3 | 0 |
| Fibromyalgia | 0 | 29 | 38 | 0 |
| Gallbladder disorder | 0 | 0 | 5 | 0 |
| Gallstone | 0 | 0 | 5 | 0 |
| Gastritis | 1 | 0 | 0 | 0 |
| Gastroesophageal reflux disease | 0 | 0 | 12 | 0 |
| Genital herpes | 0 | 0 | 1 | 0 |
| Genital warts | 0 | 0 | 3 | 0 |
| Glaucoma | 0 | 0 | 1 | 0 |
| Gluten intolerance | 1 | 0 | 0 | 0 |
| Gout | 0 | 0 | 2 | 0 |
| Hearing loss | 0 | 0 | 10 | 0 |
| Heavy metal poisoning | 1 | 0 | 0 | 0 |
| High glycolysis | 0 | 0 | 6 | 0 |
| Hypertension | 0 | 1 | 0 | 2 |
| Hypothyroidism | 1 | 2 | 0 | 1 |
| Inflammatory rheumatism | 0 | 1 | 0 | 0 |
| Irritable bowel syndrome | 3 | 2 | 13 | 0 |
| Kidney stones | 0 | 0 | 3 | 0 |
| Lyme disease | 1 | 0 | 0 | 0 |
| Lymphedema | 0 | 0 | 0 | 1 |
| Major Depression | 16 | 13 | 7 | 5 |
| Meningitis | 0 | 0 | 1 | 0 |
| Migraine | 0 | 1 | 9 | 0 |
| Minor depression | 0 | 0 | 11 | 0 |
| Multiple chemical sensitivities | 3 | 3 | 0 | 0 |
| Multiple sclerosis | 0 | 0 | 1 | 0 |
| Myalgic encephalomyelitis/Chronic fatigue syndrome | 41 | 0 | 0 | 0 |
| No other diagnosis | 0 | 0 | 0 | 11 |
| Orthostatic Intolerance | 0 | 1 | 0 | 0 |
| Osteoarthritis | 0 | 0 | 1 | 0 |
| Osteoporosis | 0 | 0 | 3 | 0 |
| Polyps | 0 | 0 | 6 | 0 |
| Post-traumatic stress disorder | 1 | 1 | 1 | 0 |
| Psoriasis | 0 | 0 | 3 | 0 |
| Renal infection | 0 | 0 | 2 | 0 |
| Restless leg syndrome | 0 | 1 | 0 | 0 |
| Sinusitis | 0 | 0 | 5 | 0 |
| Sjorden | 0 | 0 | 1 | 0 |
| Sleep apnea | 0 | 0 | 3 | 0 |
| Stenosis | 1 | 0 | 0 | 0 |
| Stomach ulcer | 0 | 0 | 9 | 0 |
| Strabismus | 0 | 0 | 2 | 0 |
| Supraventricular tachycardia | 0 | 0 | 0 | 1 |
| Tinnitus | 0 | 0 | 6 | 0 |
| Type 1 diabetes | 0 | 0 | 1 | 0 |
| Type 2 diabetes | 0 | 0 | 5 | 1 |
| Type 2 Herpes | 0 | 1 | 0 | 0 |
| Ulcerative colitis | 0 | 0 | 2 | 0 |
| Ulcerative proctitis | 0 | 0 | 0 | 1 |
| Uterine cancer | 0 | 0 | 1 | 0 |
| Uterine fibroids | 0 | 0 | 9 | 0 |
| Vertigo | 0 | 0 | 4 | 0 |
| Viral pericarditis | 0 | 1 | 0 | 0 |

Presentation of the self-reported medical diagnosis of the four groups of participants: ME/CFS patients, ME/CFS+FM patients, FM patients and HC.
